# Phytogenic Fabrication of Ag–Fe Bimetallic Nanoparticles for Cell Cycle Arrest and Apoptosis Signaling Pathways in *Candida auris* by Generating Oxidative Stress

**DOI:** 10.3390/antiox10020182

**Published:** 2021-01-27

**Authors:** Majid Rasool Kamli, Vartika Srivastava, Nahid H. Hajrah, Jamal S. M. Sabir, Arif Ali, Maqsood Ahmad Malik, Aijaz Ahmad

**Affiliations:** 1 Department of Biological Sciences, Faculty of Science, King Abdulaziz University, P.O. Box 80203, Jeddah 21589, Saudi Arabia; mkamli@kau.edu.sa (M.R.K.); nhajrah260@gmail.com (N.H.H.); jsabir2622@gmail.com (J.S.M.S.); 2 Center of Excellence in Bionanoscience Research, King Abdulaziz University, Jeddah 21589, Saudi Arabia; 3 Clinical Microbiology and Infectious Diseases, Faculty of Health Sciences, School of Pathology, University of the Witwatersrand, Johannesburg 2193, South Africa; vartika.srivastava@wits.ac.za; 4 Department of Biosciences, Jamia Millia Islamia, New Delhi 110025, India; ali.arifali@gmail.com; 5 Chemistry Department, Faculty of Science, King Abdulaziz University, P.O. Box 80203, Jeddah 21589, Saudi Arabia; 6 Infection Control Unit, Charlotte Maxeke Johannesburg Academic Hospital, National Health Laboratory Service, Johannesburg 2193, South Africa

**Keywords:** Ag–Fe NPs, *C. auris*, apoptosis, antioxidant enzymes, cell cycle

## Abstract

Novel green synthetic nanomedicines have been recognized as alternative therapies with the potential to be antifungal agents. Apoptosis induction, cell cycle arrest and activation of the antioxidant defense system in fungal cells have also gained attention as emerging drug targets. In this study, a facile and biodegradable synthetic route was developed to prepare Ag–Fe bimetallic nanoparticles using aqueous extract of *Beta vulgaris* L. Surface plasmon resonance of *Beta vulgaris*-assisted AgNPs nanoparticles was not observed in the UV-visible region of Ag–Fe bimetallic NPs, which confirms the formation of Ag–Fe nanoparticles. *Beta vulgaris-*assisted Ag–Fe NPs were characterized by FTIR spectroscopy, scanning electron microscopy, transmission electron microscopy, energy-dispersive X-ray spectroscopy, X-ray diffraction and TGA-DTG analysis for their structural and morphological properties. The as-prepared Ag–Fe NPs were well dispersed and spherical with the average particle size of 15 nm. The antifungal activity of these Ag–Fe NPs against clinical isolates of *Candida auris* was determined by broth microdilution and cell viability assays. For insights into mechanisms, induction of apoptosis and triggering cell cycle arrest were studied following standard protocols. Furthermore, analysis of antioxidant defense enzymes was determined spectrophotometrically. Antifungal susceptibility results revealed high antifungal activity with MIC values ranging from 0.19 to 0.39 µg/mL. Further studies showed that Ag–Fe NPs were able to induce apoptosis, cell cycle arrest in G2/M phase and disturbances in primary and secondary antioxidant enzymes. This study presents the potential of Ag–Fe NPs to inhibit and potentially eradicate *C. auris* by inducing apoptosis, cell cycle arrest and increased levels of oxidative stress.

## 1. Introduction

*Candida auris*, spreading across the globe and responsible for hospital outbreaks, is considered a global health threat with a high mortality rate of approximately 30–60% [1]. Due to this pathogen’s metabolic plasticity, allowing it to thrive in different habitats, frequent hospital outbreaks have been reported worldwide. Furthermore, *C. auris* isolates have been reported to be resilient to at least one antifungal class [2]. These challenges further complicate the treatment procedures, particularly in immunocompromised patient populations, who are the first targets of fungal infections. This creates an imperative need for the discovery and development of new therapeutic strategies and antimycotic drugs. Breakthroughs in multidisciplinary nanotechnology have led over the last few decades to the development of limitless engineered nanomaterials in various fields, such as catalysis, electronics, drug delivery, pollution monitoring and treatment and sensing probes [3,4,5,6,7]. The bimetallic nanomaterials are a highly interesting material class because of their possible synergistic effects, which lead to their enhanced chemical and physical characteristics, and fascinating optical, electronic and magnetic properties. Therefore, they have gained a lot of attention in scientific research and applications [8,9,10,11,12,13,14,15]. The remarkable success of nanoscience has also opened the way to creating bimetallic nanoparticles that combine monometallic nanoparticles’ properties [16]. Many researchers have stated that due to monometallic nanoparticles’ integrated functions and the two metals’ synergistic activity, bimetallic nanoparticles exhibit better properties compared to individuals [17].

Different physical and chemical approaches—for instance, co-precipitation, microemulsion, laser pyrolysis, sol–gel, laser ablation techniques and chemical reduction—have been adopted to synthesize nanoparticles [18,19,20,21]. Green synthesis of nanomaterials, which uses natural extracts such as plant extracts, microorganisms, or fermented extracts, is becoming much more popular because of its low cost and eco-friendly design [22,23]. It has many benefits, such as the fact that it can also be easily and rapidly manipulated, safety, energy efficiently and scalability [24]. Overall, biological methods eliminate the need for reactive and toxic reducing agents that harm the environment and provide more significant modulation and control over nanoparticles’ shape, size and stability [7,8,9,10]. Many reports have been presented on Fe, Au, Ag and Zn nanoparticles’ synthesis with extracts from plants and microorganisms such as fungi, yeast, bacteria and algae [24,25,26]. Biogenic nanostructure synthesis is, therefore, a safer alternative approach, since it contains non-toxic reactants derived from biological sources from unicellular organisms such as bacteria [27] and fungi [28], and extracts from plants [29,30]. Several researchers have explored many plants for bimetallic nanoparticle biosynthesis, such as the aqueous extracts of *Sago pondweed*, *Silybum marianum* and *Coleus aromaticus*, and Clove buds were used for the preparation of Au–Ag bimetallic nanoalloys with excellent catalytic and antiradical properties [31,32,33,34]. Using eucalyptus leaf extracts, both Fe nanoparticles (Fe NPs) and bimetallic Fe/Ni nanoparticles (Fe/Ni NPs) were synthesized [35]. There are many benefits to using extracts extracted from different plant parts as reducing agents for the fabrication of nanoparticles over the unicellular organism. They are readily accessible and require only easy set-ups, removing the complicated processes and maintenance of cell cultures [36]. Several reports on the synthesis of Ag–Fe bimetallic nanoparticles in the presence of palm date fruit show good in vitro antibacterial activities [37]. Ag nanoparticles produced by phytosynthetic method [38] and the production of Ag nanoparticles via green synthesis using *Beta vulgaris* peel extract [39] have been published, depicting the importance of these monometallic and bimetallic nanoparticles.

Nanoparticles are considered as an important tool in therapeutics due to their growing applications, for instance, tissue engineering, gene and drug delivery and tumor detection. As a result of small size, these particles can conveniently enter and translocate inside the cells, thereby exerting their biomedical functions. The physicochemical properties, shape, size and surface, can impact the cellular uptake, targeting and cytotoxicity of nanoparticles [40]. Mostly, the size of the nanoparticles plays a critical role in cellular uptake and determines cytotoxicity [41,42]. Most in vitro studies have demonstrated that an optimum size of 30–50 nm has a higher cellular uptake rate as compared to small (around 15–30 nm) and large (around 70–240 nm) particles, as they interact more effectively with the cell membrane receptors [41]. Small nanoparticles, ranging from several nanometers to several hundreds of nanometers are internalized by mechanism of pino- or micropinocytosis, whereas particles ranging from 250 nm to 3 μm have displayed efficient in vitro phagocytosis. Furthermore, nanoparticles ranging from 120 to 150 nm enter via clathrin- or caveolin-mediated endocytosis, and even larger (200 nm) nanoparticles also employ the same pathway [43,44]. Additionally, the spherical nanoparticles have five-times higher cellular uptake compared to rod-shaped nanoparticles [41]. However, not much has been reported in the literature regarding cellular uptake of Ag–Fe nanoparticles specifically in *Candida* cells, but the above literature very well supports our curiosity to check the toxicity of newly synthesized Ag–Fe nanoparticles against *C. auris* strains.

The mainline defense against fungi is the innate immune system in human hosts, which activates the intracellular signaling cascade within fungal cells. Consequently, generating excess reactive oxygen species (ROS) causes cell damage and eventually cell death [45]. Aerobic organisms produce ROS at lower or moderate rates as the crucial by-products of normal biochemical processes. However, increasing ROS levels are kept in check by a dynamic antioxidant defense mechanism, which in turn helps establish an infection in host cells. In *C. albicans*, upregulation of the regulatory *CAP1* gene mediates the production of vital antioxidant enzymes (catalase, superoxide dismutase, glutathione peroxidise, glutathione reductase and glutathione transferase) to repair the damage caused by host oxidative stress [37,38,45,46]. However, an imbalance between antioxidative enzymes and cellular ROS levels can be harmful. It can trigger oxidative stress within *Candida* cells, resulting in cellular apoptosis and cell cycle arrest [47]. The role of antioxidant enzymes in combating oxidative stress is well studied in *C. albicans*; however, there is no study reporting the antioxidant enzymes in *C. auris*.

Researchers have established the antifungal activity of nanoparticles less than 5 nm across (gold, silver and gold-silver nanoparticles) against five *Candida* species [48], and other bimetallic Fe–Ag and Fe–Cu nanoparticles were reported to be active against various species of bacteria and *Candida* [49,50]. We synthesized a new Ag–Fe nanoparticle (Ag–Fe NPs) and evaluated their anti-candidal effects against different clinical *C. auris* isolates with the above background. To further understand the in-depth antifungal mode of the antifungal action of Ag–Fe NPs, induction of apoptosis, the effect on oxidative stress enzymes and cell cycle arrest were studied in *C. auris*.

## 2. Materials and Methods

### 2.1. Chemicals

Analytical grade metal precursors (silver nitrate (AgNO_3_) 99% (molecular weight: 169.87 g/mol), iron (III) nitrate (Fe(NO_3_)_3_) 99%) (molecular weight 241.86 g/mol), were purchased from Sigma-Aldrich Co. (St Louis, MO, USA). H_2_O_2_ (10 mM; Merck, Darmstadt, Germany), ascorbic acid (500 mg/mL; Sigma Aldrich Co., St. Louis, MO, USA). *C. auris* strains (*n* = 25) procured from NICD, South Africa. All the reagents were used as received without performing any further purifications. All the metal precursors’ stock solutions were prepared in a clean environment using deionized, double distilled water as a solvent.

### 2.2. Collection and Preparation of the Beta vulgaris L. Extract

Fresh *Beta vulgaris* L., commonly known as beetroot, was collected from the local vegetable market in Jeddah, Saudi Arabia. The beetroot was washed properly with tap water to remove all soil particles from the surface, followed by several washings with double distilled water. The cleaned beetroot was sliced expertly into small pieces and dispersed into a conical flask containing 250 mL of double-distilled water and heated for 30 min at 70 °C. The acquired extract was cooled at room temperature and centrifuged for 10 min. At room temperature, the beetroot extract was cooled and filtered with Whatman No. 1 Filter Paper, centrifuged for 30 min at 7000 rpm to remove dense biomaterial, filtered and stored before further experimental use in a refrigerator at 4 °C.

### 2.3. Preparation of Ag–Fe NPs

The phytogenic green synthesis of Ag–Fe bimetallic nanoparticles was carried out according to the previously reported protocol [25]. In this synthesis process, 25 mL of 2 mM AgNO_3_ was mixed with 2 mM Fe(NO_3_)_3_ in a three-neck flask with constant stirring and room temperature (25 °C). After that, 25 mL of freshly prepared beetroot extract was added to the above-prepared metal precursor solution under constant stirring with a magnetic stirrer at 25 °C. The transformation of the reaction color recognized the synthesis of Ag–Fe nanoparticles—ruby red or purple. Finally, the Ag–Fe nanoparticles were separated by centrifugation of the colored solution for 30 min at a speed of 10,000 rpm. The acquired solid material was washed with distilled water and ethanol multiple times, followed by drying in a vacuum oven at 100 °C. The solid material was subsequently used for further characterizations and applications.

### 2.4. Characterization of Ag–Fe Bimetallic NPs

The optical properties of Ag–Fe NPs were recorded on a UV–Visible spectrophotometer (Thermo Scientific Evolution 600 UV-Vis spectrophotometer) in the wavelength range of 200–800 nm using a quartz cuvette of 10 mm path length. The FTIR spectra of the freshly prepared beetroot extract and as-synthesized Ag–Fe nanoparticles were recorded on ATR FTIR spectrophotometer (Bruker FTIR spectrophotometer (Model: ALPHA II, Bruker, Billerica, MA, USA) at a resolution of 4 cm^−1^ in 450 −4000 cm^−1^ in transmission mode to determine the active involvement of the biomolecules in the synthesis and stabilization of nanoparticles. An X-ray diffraction spectrometer (XRD Bruker, D8 advanced, Bruker, Germany), fitted with a CuKα source (λ = 1.54060 Å), has been used to validate crystalline planes and crystallinity for green-synthesized Ag–Fe nanoparticles. A normal quantity of Ag–Fe nanoparticles was dispersed on a clean sample holder to create a thick film for spectral analysis with a range of 20–80° θ angle, which was carried out at room temperature. Transmission electron microscopy (JEOL JEM-2100F Field emission microscopy, Akishima, Tokyo, Japan) operated at 200 kV, and field-emission scanning electron microscopy (FEG-SEM: Zeiss 540 ultra) operated at 15.0 kV accelerating voltage equipped with energy dispersion were used to investigate the size, morphology, elemental analysis and crystallization of Ag–Fe bimetallic NPs. Thermogravimetric analysis (TGA) of the as-prepared Ag–Fe bimetallic nanoparticles using aqueous extract of beetroot was carried out by using a Perkin Elmer, Pyris Diamond instrument with a heating rate of 10 °C min^−1^, under a nitrogen atmosphere.

### 2.5. Biological Assays

*Candida auris* strains (*n* = 25) procured from the NICD, South Africa, and preserved in the department were used in the present study. Ethical approval for using clinical strains for experimental purposes was granted by Wits Human Research Ethics Committee (M140159).

#### 2.5.1. Antifungal Activity of Ag–Fe Nanoparticles (Ag–Fe NPs)

Antifungal susceptibility testing for Ag–Fe NPs was performed by broth microdilution assay against 25 clinical strains of *C. auris*, following the Clinical and Laboratory Standards Institute guidelines [51]. The concentration ranges of Ag–Fe NPs and amphotericin B (positive control) used were 12.5–0.02 µg/mL and 16–0.031 µg/mL, respectively. Furthermore, negative (1% DMSO), culture and medium controls were included in the experiment. MIC values were recorded on the basis of visual observations.

The Minimum Fungicidal Concentration (MFC) values for Ag–Fe NPs were assessed by sub-culturing 10 µL from wells showing no growth on Sabouraud dextrose agar (SDA) plates. Results were recorded after incubating the plates at 37 °C for 24 h.

#### 2.5.2. Agar Diffusion Assay

To further determine the susceptibility of *C. auris* against Ag–Fe NPs, a standard agar diffusion assay was done following the protocol described previously [52]. Briefly, 0.5 MacFarland of *C. auris* MRL6057 was inoculated in molten agar media at 40 °C, poured in a 90 mm Petri dish and allowed to solidify at room temperature. After solidification, sterile filter discs impregnated with 1% DMSO (negative control), 2 µg/mL amphotericin B, 0.39 µg/mL (MIC) and 0.78 µg/mL (MFC) were placed on agar plates. The plates were allowed to dry at room temperature, followed by an incubation at 37 °C for 48 h. After incubation, diameters of zones of inhibition (ZOIs) were recorded in millimeters around all the discs.

#### 2.5.3. Cell Count and Viability

The fungicidal activity of Ag–Fe NPs was quantified by cell count and viability assay using Muse^TM^ Count and Viability assay kit, following the manufacturer’s instructions. *C. auris* MRL6057 cells were grown and exposed to Minimum Inhibitory Concentration (MC), ½ MIC and MFC values of Ag–Fe NPs and incubated at 37 °C for 4 h. After that, exposed yeast cells were washed, and an aliquot of 20 µL was mixed with 380 µL of Count and Viability reagent, followed by 5 min incubation at room temperature. Muse^TM^ Cell Analyzer examined the cell count and viability of treated and untreated yeast cells. The yeast cells exposed to H_2_O_2_ (10 mM; Merck, Germany) were taken as a positive control, whereas healthy untreated cells were considered a negative control.

### 2.6. Apoptotic Studies

#### 2.6.1. Protoplast Preparation

According to the method described previously, protoplasts were prepared from *C. auris* MRL6057 cells [53]. *C. auris* cells in mid-log phase were exposed to ½ MIC, MIC and MFC values of Ag–Fe NPs for 4 h at 37 °C and were treated with protoplast buffers 1, 2 and 3. Next, protoplasts were precipitated at 1500 rpm for 5 min. They were resuspended gently with sterile PBS until further use.

#### 2.6.2. Effect on *C. auris* Mitochondrial Membrane Potential (Δψm)

The effect of Ag–Fe NPs on *C. auris* MRL6057 Δψm was gauged by JC-10 mitochondrial membrane potential assay kit (Abcam, Cambridge, UK); manufacturer’s instructions were strictly followed. The green fluorescence (X) was recorded at Ex/Em = 490/530 nm, whereas red fluorescence (Y) was recorded at Ex/Em = 540/590 nm, using SpectraMax iD3 multi-mode microplate readers (Molecular Devices, San Jose, CA, USA). The difference in mitochondrial membrane potential was gauged as the ratio of JC-10 aggregates (Y mean) and JC-10 monomeric (X mean) forms. *C. auris* cells exposed to H_2_O_2_ (10 mM) were taken as a positive control, whereas healthy untreated cells were considered the negative control.

#### 2.6.3. Effect on Cytochrome c Discharge

The cytochrome c discharge after exposure to ½ MIC, MIC and MFC values of Ag–Fe NPs for 4 h at 37 °C was examined as described previously [54]. Cytochrome c, both cytosolic and mitochondrial, was recorded at 550 nm using SHIMADZU spectrophotometer.

#### 2.6.4. Phosphatidylserine Externalization

The phosphatidylserine (PS) exposure in the cell membrane indicates the initial phase of apoptosis. Therefore, the transfer of PS from inside to outside of the cell membrane was evaluated using an Apoptosis Kit (Thermo, Bremen, Germany), as instructed by the manufacturer. A flow cytometer (BD Biosciences, San Jose, CA, USA) was used for the study, and for result analysis FlowJo_V10 software was used. Quadrants 1, 2, 3 and 4 respectively represent necrosis, late apoptosis, early apoptosis and live cells.

### 2.7. Cell Cycle Arrest

To study the magnitude of Ag–Fe NPs on the cell cycle, Muse^TM^ Cell Analyzer was used. The Muse™ Cell Cycle kit was used for this experiment, and instructions given by the manufacturer were followed. Briefly, yeast cells were propagated till mid-log phase, later spun at 3000 rpm for 4 min, suspended in SDB (0.5 McFarland, MI, USA) and exposed to ½ MIC, MIC and MFC of Ag–Fe NPs for 4 h. The cells were washed with PBS, and the pellet was secured and fixed in 70% chilled ethanol (1 mL; Sigma Aldrich Co., St. Louis, MO, USA). The fixed cells (200 µL) were again centrifuged and washed with fresh PBS. Muse™ Cell Cycle reagent (200 µL) was added to the fixed cells and incubated for 30 min in the dark. Both positive and negative controls were given the same treatment.

### 2.8. Antioxidant Enzymes Assays

To study the effect of Ag–Fe NPs on the antioxidant enzymes, *C. auris* MRL6057 cells were exposed to (½ MIC, MIC and MFC of Ag–Fe NPs for 4 h, followed by exposure of cells to Ag–Fe NPs. Cell free extracts (CFE) were synthesized as described previously [55] and were used for the analysis of antioxidant enzymes and estimation of lipid peroxidation (LPO).

#### 2.8.1. Catalase (CAT)

CAT activity was determined spectrophotometrically by the procedure described previously [55]. The utilization of hydrogen peroxide was measured every 30 s for 3 min, and CAT activity was calculated as hydrogen peroxide consumed per minute in µmol, using an extinction coefficient of 0.081 × 10^−1^/mM/cm.

#### 2.8.2. Superoxide Dismutase (SOD)

SOD activity was determined spectrophotometrically by following the procedures described by [55]. The enzyme activity was measured using pyrogallol autoxidation inhibition for 3 min every 30 s.

#### 2.8.3. Glutathione Peroxidase (GPx)

The enzyme activity was spectrophotometrically estimated by the procedures described previously [56]. GPx activity was calculated as NADPH oxidized per minute in µmol, using an extinction coefficient of 6.22 × 10^3^/M/cm.

#### 2.8.4. Glutathione Reductase (GLR)

The enzyme activity was spectrophotometrically measured by following the previously mentioned protocol [56]. The enzyme activity was determined as NADPH oxidized per minute in µmol, using an extinction coefficient of 6.22 × 10^3^/M/cm.

#### 2.8.5. Glutathione Transferase (GST)

The activity was measured spectrophotometrically as described by Maras et al. [57]. The activity was evaluated as a GSH-CDNB conjugation rate, readings taken after 30 s for 3 min at 340 nm. The GST activity result is expressed as 1 µmol glutathione conjugated per minute.

### 2.9. Lipid Peroxidation (LPO)

The thiobarbituric acid reactive substances (TBARS) method was used for the analysis of lipid peroxidation, and the protocol was adopted from Khan et al. [55]. The pellet secured and preserved during CFE preparation containing plasma membrane was resuspended in Tris-HCl buffer with pH 7. An aliquot (200 μL) was added to PBS (1.8 mL; pH 7) and incubated for an hour at 37 °C. Post-incubation, the reaction was stopped by adding 10% TCA and 0.67% TBA, followed by boiling the tubes for 20 min, cooling on ice and spinning at 2500 g for 10 min. LPO was calculated by measuring OD at 432 nm, using a molar extinction coefficient of 1.56 × 10^5^/M/cm, and the results are expressed as nmol of TBARS formed.

### 2.10. Cytotoxicity Assay

The cytotoxic effect of Ag–Fe NPs (½ MIC, MIC, and MFC) was evaluated in terms of percent hemolysis, using horse red blood cells (NHLS, Sandringham, South Africa) as described by Lone et al. [53]. The positive control was maintained using 1% Triton X-100, whereas fresh PBS was used as a negative control. The percentage of hemolysis was calculated using the formula mentioned below:

(1) $$\%\;Haemolysis\;=\;\frac{\left[\left(A450\;of\;treated\;sample\right)\;-\;\left(A450\;of\;negative\;control\right)\right]}{\left[\left(A450\;of\;positive\;control\right)\;-\;\left(A450\;of\;negative\;control\right)\right]}\;\times \;100$$

### 2.11. Statistics

All the experiments were done in triplicate and the results are represented as means ± standard errors. The data were analyzed using GraphPad Prism 8.0.1, using two-way ANOVA. *p-*values ≤ 0.05 were considered significant.

## 3. Results and Discussion

### 3.1. Chemistry

#### 3.1.1. UV–Visible and FTIR Spectroscopic Analysis of Ag–Fe NPs

**Figure 1:**
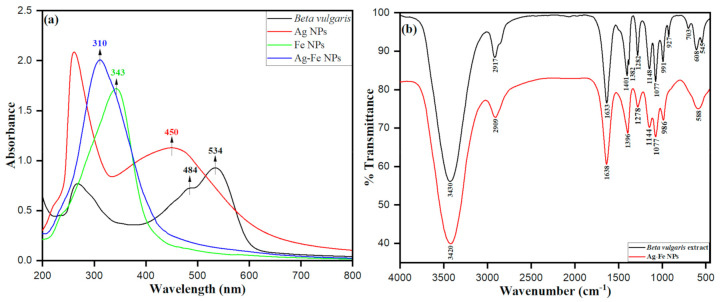
(**a**) UV–visible spectra of *Beta vulgaris* extract, AgNPs, FeNPs and Ag–Fe NPs; (**b**) FTIR of biosynthesized Ag–Fe bimetallic nanoparticles from *Beta vulgaris* L. extract.

The biosynthesis of Ag–Fe nanoparticles was deduced from *Beta vulgaris* L. extract as inferred from the obtained UV–vis spectra depicted in Figure 1a. The observed intensity peaks at λ_max_ ca. 484 nm and ca. 534 nm of *Beta vulgaris* L. extract are from the characteristic pigment adsorption intensities corresponding to yellow betaxanthin and red–purple betanin, respectively [3]. The possible biosynthesis of Ag–Fe nanoparticles was physically observed in the color change from light yellow to dark brown upon the addition of *Beta vulgaris* L. extract to the mixture. The observed intensity peak at ca. 310 nm was achieved by the biosynthesized Ag–Fe nanoparticles. The counter monometallic Ag nanoparticles showed a surface plasmon resonance (SPR) peak at ca. 450 nm, and Fe nanoparticles showed an absorption peak at ca. 343 nm. Upon the addition of the aqueous extract of *Beta vulgaris* into the Fe^3+^ and Ag^+^ ions aqueous solution, the SPR peak at 450 nm drastically disappeared and a peak (343 nm) corresponding to Fe NP showed an increase in peak intensity with a plasmon shifted to 310 nm and broadening of absorption band for Ag–Fe NPs, indicating the formation of stable Ag–Fe nanoparticles.

The presence of various functional groups of the phytochemical persuation, including pigments such as betanin, isobetanin, vulgaxanthin I and II, flanovids, phenolic amides and phenolic acids in *Beta vulgaris* L. extract, was determined from the FTIR spectra; they were apparent after the possible biosynthesis of Ag–Fe bimetallic nanoparticles. These phytochemicals and pigments participate in the bio-reduction of Ag^+^ ions into Ag^0^ and Fe^3+^ into Fe^0^ as capping/stabilizing agents. Consequently, the various stretching vibrations at 3420, 2909, 1638, 1396, 1278, 1077, 986 and 588 cm^−1^ were observed in the FTIR spectra of Ag–Fe nanoparticles. The FTIR spectrum of biosynthesized Ag–Fe bimetallic nanoparticles from *Beta vulgaris* L. extract possesses various stretching vibrations within the range of Ag–Fe nanoparticles, as depicted in Figure 1b. The obtained intense peak at v ≈ 3430 cm^−1^ within the transmission range 3600–3200 cm^−1^ was attributed to the presence of phenolic hydroxyl functional group –OH stretching vibrations. It is worth mentioning that the presence of phenolic groups involved in proton dissociation helps with attaining the reduction of the metal ion in the reaction mixture. The peak appeared in the range between v ≈ 3000 and 2850, i.e., v ≈ 2917 cm^−1^, apparently after C-H stretching vibrations in alkenes. The peak intensity at 1633 cm^−1^ within transmission range v ≈ 1680–1620 cm^−1^ was attributed to the stretching vibration of C=O in aldehydes, ketones or even amides. Observed frequencies such as 1401, 1382 and 1282 cm^−1^ are within frequency range v ≈ 1800–1200 cm^−1^, corresponding to the carbonyl (−C=O) stretching vibrations. The peak intensity at v ≈ 1148 cm^−1^ was attributed to the –C–N stretching vibration of aliphatic amines. The peak at v ≈ 1077 cm^−1^ corresponds to the presence of C–O–C symmetric and asymmetric starching vibrations. However, the lower intensity peaks apparent at 608 and 542 cm^−1^ are attributable to specifically the biosynthesis of bimetallic Ag–Fe nanoparticles facilitated by betanin of *Beta vulgaris* L. extract.

#### 3.1.2. XRD Analysis of Biosynthesized Ag–Fe Nanoparticles from *Beta vulgaris* L. Extract

**Figure 2:**
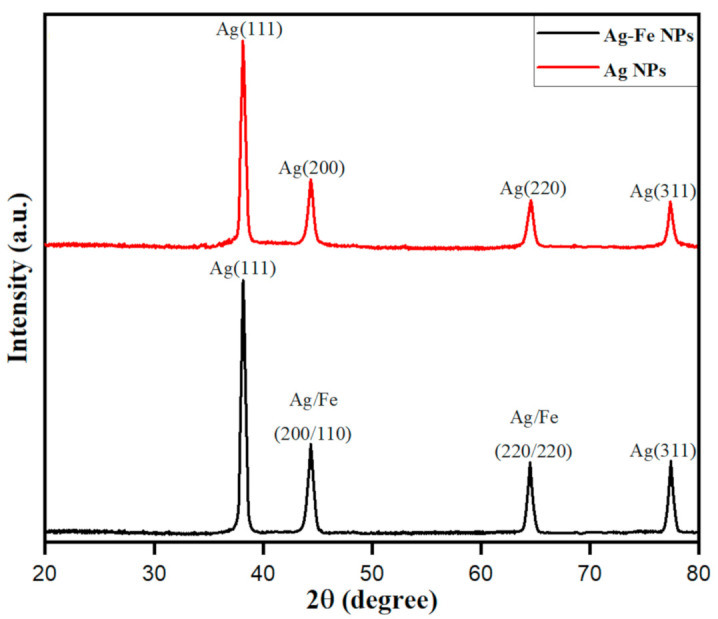
XRD pattern of the AgNPs and Ag–Fe NPs.

The XRD analysis was done to deduce the crystalline nature with the lattice properties of biosynthesized Ag–Fe nanoparticles from *Beta vulgaris* L. extract, as shown in Figure 2. The XRD diffraction peaks were obtained, and the particle size was calculated by using the Debye–Scherrer equation as:

(2) $$D_{}{=}_{}\frac{K_{}\lambda }{\beta \cos\theta }$$

where *D* represents the average crystallite size, *K* is the Scherrer constant (0.94), *λ* is the wavelength of X-ray sources (0.15406 nm), *β* is the full width at half maximum in radians (FWHM) and *θ* is the diffraction angle in radians. By utilizing each term’s numerical values in Equation (1), the average particle size of biosynthesized Ag–Fe nanoparticles from *Beta vulgaris* L. extract was 15.65 nm. Besides, the crystalline nature of Ag–Fe nanoparticles was deduced from 2*θ* values of major diffraction peaks at 38.01, 44.21, 64.7 and 77.49° with lattice planes (111), (200), (220) and (311) respectively, as is crystal clear in Figure 2. The results of XRD patterns show a face-centered cubic (fcc) phase for Ag particles, according to the JCPDS number 00-004-0783. However, Fe shows diffraction peaks at 2*θ* values 44.21 and 64.7° with lattice planes (110) and (220), and the absence of other peaks in the XRD pattern reflects that the Fe nanoparticles are protected from oxidation and are possibly reduced onto the surface of Ag in Ag–Fe nanoparticles biosynthesized from *Beta vulgaris* L. extract. 

#### 3.1.3. Morphological Analysis of Ag–Fe NPs

**Figure 3:**
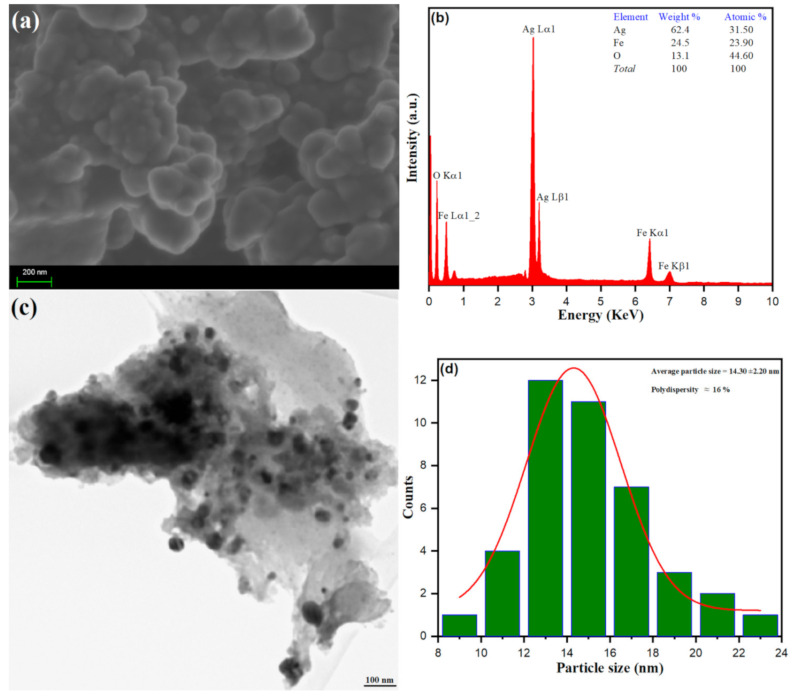
(**a**) SEM, (**b**) EDX (**c**) TEM and (**d**) particle size distribution of Ag–Fe NPs.

The morphology, size and elemental composition of the Ag–Fe from *Beta vulgaris* L. extract was perceived using characterization via SEM, EDX and TEM, and particle size distribution analysis, as depicted in Figure 3. Surface morphology from SEM analysis was established; the spherical bunches of nanoparticles with forth like projections in an aggregation manner were observed, as shown in Figure 3a. The chemical composition of biosynthesized Ag–Fe from Beta vulgaris L. extract was securitized upon EDX analysis, as depicted in Figure 3b. The observed peaks at 3 keV (Ag), 6 keV (Fe), 6.6 keV (Fe), and 0.5 keV (O) from the EDX spectrum advanced the biosynthesis of Ag–Fe nanoparticles. In our experimental conditions, the complete oxidation of Fe was restricted to its different oxidation forms. However, the O peak in EDX confirmed some of the Fe molecules are oxidized to their corresponding oxides. Besides, the high-resolution transmission electron microscopy (TEM) analysis further deduced the presence of spherical shaped biosynthesized Ag–Fe nanoparticles within a range of 100 nm. However, TEM analysis of Ag–Fe nanoparticles from *Beta vulgaris* L. extract, as depicted in Figure 3c, shows an agglomeration of nano-spheres with varying sizes. The average particle size distribution was calculated using image software, as shown in Figure 3d. The average particle size of biosynthesized Ag–Fe nanoparticles of spherical morphology was calculated to be 14.30 ± 2.20 nm with 16% polydispersity. The results obtained from the morphological analysis further emphasize the biosynthesis of Ag–Fe nanoparticles from *Beta vulgaris* L. extract.

The cellular uptake and biological reactions may be controlled by the agglomeration state of nanoparticles, and there is adequate evidence that interactions of the Ag nanoparticles with biomolecules may result in particle aggregation [58,59]. Several studies have reported easy penetration of agglomerated Ag nanoparticles in mesenchymal stems cells and nuclei, and lower cytotoxicity has also been reported in agglomerated forms as compared to free AgNPs [58,60]. Therefore, due to higher cellular uptake of newly synthesized Ag–Fe nanoparticles (average size, 14.30 ± 2.20 nm and spherical shape), they can exert a strong fungicidal effect against pathogenic *C. auris* strains.

#### 3.1.4. TGA/DTG Analysis of Ag–Fe NPs

**Figure 4:**
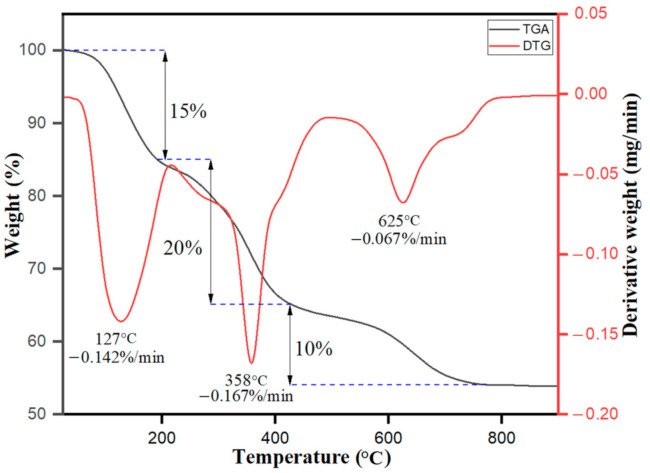
TGA-DTG curves for Ag-Fe NPs.

The thermogravimetric and derivative thermogravimetric analysis (TGA/DTG) of the biosynthesized Ag–Fe nanoparticles from *Beta vulgaris* L. extract was conquered, as shown in Figure 4. The results obtained from the data show sample weight loss in different stages—i.e., below 200 °C with DTG peak intensity at 127 °C with derivate weight rate at −0.142% per min attributed to the weight loss of 15% because of evaporation of moisture content in the sample due to adsorption of water vapors from surroundings. After the initial stage, the biosynthesized nanoparticles firmly maintained their surface structure. The next step occurred from 200 °C to 270 °C was the transition phase change with a weight loss of about 20% due to thermal degradation of volatile phytochemicals acting as the reducing agents on the surfaces of Ag–Fe nanoparticles. Besides the increase in temperature, further weight loss (about 10%) occurred in the temperature range from 270 °C to 420 °C with a DTG hump at 358 °C and a derivate weight rate of −0.167% min attributable to the thermal decomposition of the organic skeleton—breakdown of hydrocarbon chains. The further increase in temperature showed the crystal phase change from 420 °C to 800 °C with a minor DTG hump at 625 °C and a derivate weight rate of −0.067% per min; that was all observed due to the mass loss of the final carbon-based moieties being evaporated. The overall results and interpretation as acquired from the TGA/DTG curve data further support the successful biosynthesis of Ag–Fe nanoparticles from *Beta vulgaris* L. extract.

### 3.2. Biology

#### 3.2.1. Anti-Candida Activity of Ag–Fe NPs

Various concentrations of Ag–Fe NPs were used to evaluate MIC and MFC against different clinical *C. auris* isolates. The MIC values were found to range from 0.19 to 0.39 µg/mL, whereas MFC was recorded from 0.39 to 0.78 µg/mL (Table 1). For the positive control, MIC and MFC values were found between 0.125 and 4 µg/mL and 0.25 and 8.0 µg/mL respectively (Table 1).

Ag NPs have already been known to have antifungal activity against *C. albicans*, both alone and in combination with antifungal drugs. A recent study has reported the strong inhibitory effect of Ag NPs against *C. albicans* at a concentration of 1.8 mg/mL when combined with cationic carboxilane [61]. Another study, wherein they capped Ag NPs with L-3,4-dihydroxyphenyl-alanine, reported reducing MFC values to 31.2–62.5 μg/mL [62]. In a separate study, it has been reported that iron oxide (Fe_2_O_3_) NPs inhibit the growth of different fungal pathogens in a concentration range of 0.063–0.016 mg/mL [63]. It has also been reported that combinations of metals are of high interest in nanomedicine in comparison to the corresponding monometallic nanoparticles [64]. All these studies stand in strong support of our results, which are that bimetallic Ag–Fe NPs show high antifungal activity against MDR *C. auris* strains. Based on the low MIC and MFC values, we further quantified these nanoparticles’ inhibitory effect using the cell viability assay. Additionally, based on the MIC results, *C. auris* MRL6057 was selected as a representative strain for further in-depth studies.

#### 3.2.2. Agar Diffusion Assay

Agar diffusion assay results indicated fungicidal activity of Ag–Fe NPs against *C. auris*, as is evident from the clear zones of inhibition around the discs impregnated by MIC and MFC values of the Ag–Fe NPs (Supplementary Materials Figure S1). The mean diameters of ZOIs around the disc with MIC and MFC values were 17 and 22 mm. The disc impregnated with 1% DMSO (solvent control) showed no ZOI, thereby supporting that 1% DMSO has no antifungal activity. Interestingly, the discs with 2 µg/mL amphoterecin B had very faint and small ZOIs, which further indicated that *C. auris* MRL6057 is resistant to amphoterecin B.

#### 3.2.3. Cell Count and Viability Assay

**Figure 5:**
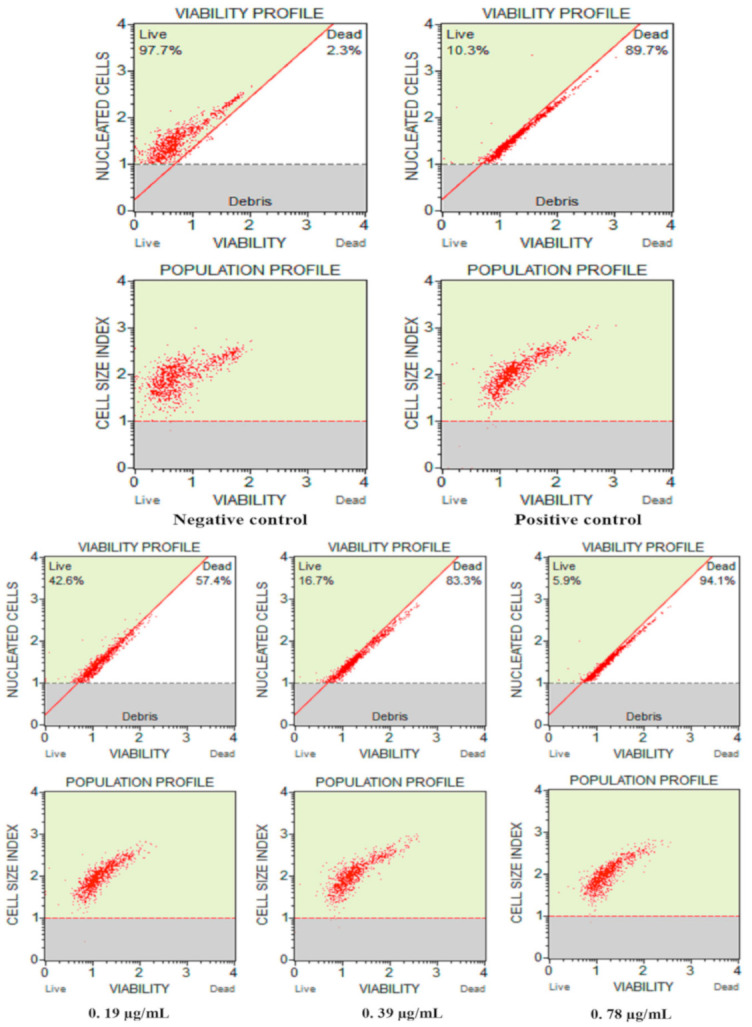
Effects of Ag–Fe NPs on the viability and cell count of *C. auris* MRL6057. Untreated and cells treated with 10 mM H_2_O_2_ represent negative and positive controls respectively. For treatment, *C. auris* cells were exposed to different concentrations of Ag–Fe NPs.

The population profile and viability of *C. auris* after exposure to different Ag–Fe NPs concentrations are represented in Figure 5. The negative control showed healthy growing cells—97.7% live cells, whereas in the positive control (H_2_O_2_) only 10.3% of cells were alive. A dose-dependent decrease in the percentage of viable *C. auris* cells was recorded after exposure to the Ag–Fe NPs. The cell viability at concentrations of ½ MIC, MIC and MFC of Ag–Fe NPs were 42.6%, 16.7%, and 5.9%, respectively. These results ascertained that Ag–Fe NPs at their MFC value completely inhibit the growth and survival of *C. auris* MRL6057, which validates Ag–Fe NPs’ anti-*Candida* potency at their MIC and MFC values.

Ag NPs have demonstrated significant antimicrobial properties against both bacterial and fungal pathogens [65]. Furthermore, Fe NPs have also been reported to possess both antibacterial and antifungal activities [63,66]. These studies further support our claims that Ag–Fe NPs have intense anti-*Candida* activity, which could be a synergistic interaction between these two metals. The ability of metallic nanoparticles to inhibit the growth and viability of pathogens is presumably the result of various effects, including their encounters with cells resulting in compromised membrane permeability, cell disruption, ROS formation, damage to cellular DNA and RNA, microbial cell lysis and inactivation of crucial enzymes [66,67,68].

#### 3.2.4. Apoptotic Studies

- Mitochondrial membrane potential

**Figure 6:**
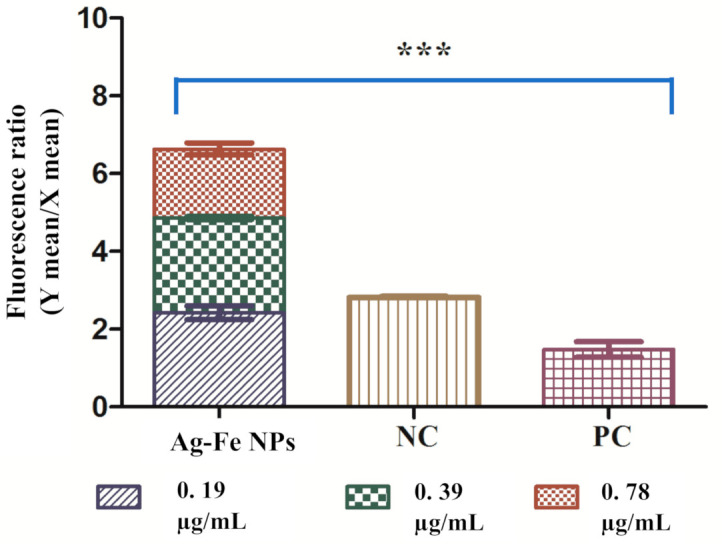
Decrease in mitochondrial membrane potential in *C. auris* cells exposed to different concentrations of Ag–Fe NPs. Untreated cells represent the negative control (NC), and cells treated with 10 mM H_2_O_2_ represent the positive control (PC). *** *p* ≤ 0.05.

To study the physiology of cell death caused by Ag–Fe NPs, we investigated mitochondrial membrane potential (Δψm) by determining JC-10 aggregates: JC-10 monomers. A reduction in this ratio between treated and untreated control indicated depolarization of Δψm. The live yeast cells have a stable Δψm; aggregates of JC-10 dye are formed with red fluoresces. Apoptotic cells have decreased Δψm with the monomeric form of dye which produces green fluorescence. Compared to the negative control, a noteworthy increase in JC-10 monomers was observed in the treated samples, suggesting depolarization. The results for Ag–Fe NPs-treated and untreated yeast cells are represented in Figure 6. The ratio in the negative control was 2.82, which lowered to 1.47 in positive control cells. In the case of Ag–Fe NPs exposure, maximum depolarization was seen at a MFC value with a ratio of 1.77, whereas at ½ MIC and MIC values, the ratios were 2.43 and 2.42 respectively.

Mitochondria are important for cell survival and play a role in apoptosis; consequently, loss of Δψm is considered a necessary step of the apoptotic pathway. Based on the results, Ag–Fe NPs causes disintegration of the mitochondrial membrane in *C. auris* cells by reducing the mitochondrial membrane potential. The depolarization results from unregulated mitochondrial membrane pores and therefore causes movement and triggers different pro-apoptotic factors. This feature is observed during early apoptosis and is associated with cytochrome c release. Ag NPs have been found to induce mitochondrial toxicity, which can be identified as loss of membrane potential, changes in calcium sequestration and inhibition of oxidative phosphorylation [69]. Furthermore, Hwang and co-workers also reported mitochondrial membrane depolarization after the apoptosis induction by Ag NPs in *C. albicans* [70]. In a separate study, different Fe NPs were also reported to cause apoptotic cell death in cultured human umbilical endothelial cells by disrupting mitochondrial membrane potential [71]. These studies are endorsing our results; hence, we hypothesize that both these metals in bimetallic NPs are causing mitochondrial membrane depolarization in *C. auris* and resulting in apoptotic cell death. However, further comparative studies between the monometallic and bimetallic NPs will be required to authenticate these claims.

- Cytochrome c release

**Figure 7:**
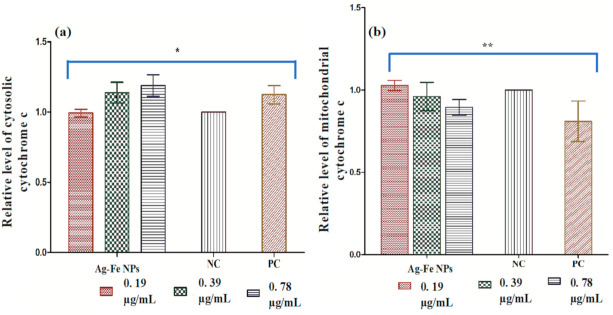
Activation of apoptotic factors in the *C. auris* MRL 6057 response to Ag–Fe NPs and H_2_O_2_ (10 mM). (**a**,**b**) represent mitochondrial and cytosolic cytochrome c respectively. NC: negative control; PC: positive control. Ag–Fe NPs triggers PS externalization in *C. auris.* * *p* ≤ 0.001; ** *p* ≤ 0.005.

Another important parameter of apoptosis in yeast cells is the release of cytochrome c from the mitochondria to the cytosol. Ag–Fe NPs resulted in decreased mitochondrial cytochrome c, and the cytosolic cytochrome c level was found elevated compared to the healthy untreated control (Figure 7a,b). The release of cytochrome c was moreover directly proportional to the concentration of Ag–Fe NPs. The recorded values for mitochondrial and cytosolic cytochrome c levels were set as 1.0. The relative values of mitochondrial and cytosolic cytochrome c in the case of the positive control were 0.8 and 1.12, respectively. A maximum discharge was recorded at a concentration of 0.78 µg/mL with the relative values 0.89 and 1.18 for mitochondrial and cytosolic cytochrome c, respectively. Similarly, the values recorded for mitochondrial and cytosolic cytochrome c after exposure to 0.39 µg/mL of Ag–Fe NPs were 0.96 and 1.13, respectively. However, at the lower concentration of 0.19 µg/mL, the values were recorded as 1.02 and 0.99 respectively, for mitochondrial and cytosolic cytochrome c—very close to the negative control.

Cytochrome c is an electron transfer center in mitochondria and plays a vital role in apoptosis. Therefore, estimating its discharge from mitochondria to the cytosol clearly demonstrates the proper functioning of the electron transport chain [72]. Release of cytochrome c from mitochondria to cytosol is directly linked to apoptosis. Our results depicted that Ag–Fe NPs affect the electron transport chain and cause apoptosis in *C. auris* cells. Overall, in the present study, Ag–Fe NPs destabilized the mitochondrial membrane and released cytochrome c into the cytosol. Release of cytochrome c thereby activated yeast metacaspase Yca1p, which triggered caspase cascade-mediated apoptosis in *Candida* cells [73]. Several other studies also reported that mitochondrial dysfunction and discharging cytochrome c into the cytosol could exert cellular apoptosis in *Candida* species [53,70,74].

**Figure 8:**
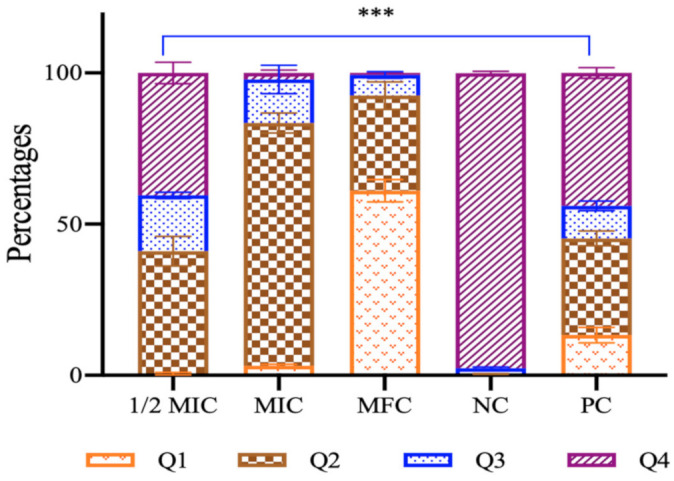
Phosphatidylserine exposure in *C. auris.* Cells were exposed to 0.19 µg/mL (½ Minimum Inhibitory Concentration - MIC), 0.39 µg/mL (MIC) and 0.78 µg/mL (Minimum Fungicidal concentration—MFC) of Ag–Fe NPs. NC represents untreated negative control and PC represents H_2_O_2_ treated positive control. *** *p* ≤ 0.05.

The annexin V and PI double staining assay was used to evaluate the most studied apoptotic marker, PS externalization, in *C. auris* MRL6057 cells. The membrane integrity was corroborated by PI staining, whereas exposed PS was checked by annexin V staining, permitting demarcation between different apoptotic and necrotic cells. At different sub-inhibitory concentrations, the percentages of cells in the Q1 (annexin V^−^/PI^+^), Q2 (annexin V^+^/PI^+^) and Q3 (annexin V^+^/PI^−^) quadrants increased. In contrast, in the Q4 (annexin V^−^/PI^−^) quadrant, the percentage of live cells decreased significantly (Figure 8). As expected, the negative control had 97.6% viable cells in Q4; and for the positive control, the cells were distributed in all the quadrants with 13.3% in Q1, 32% in Q2, 10.8% in Q3 and 43.9% in Q4, suggesting necrosis and late apoptosis in most of the cells. Higher concentrations of the Ag–Fe NPs resulted in a higher percentage of cells confined to Q1 and Q2. At 0.78 µg/mL, we observed 61.1%, 31.5%, 6.9% and 0.7% of cells in Q1, Q2, Q3 and Q4 respectively. At 0.39 µg/mL, 3.2%, 80.3%, 14.4% and 2.2% of cells were in Q1, Q2, Q3 and Q4 respectively; and at 0.19 µg/mL, 0.6%, 38.5%, 18.5% and 42% of cells were in Q1, Q2, Q3 and Q4 respectively. Our results are clearly showing a decrease in cell percentage in Q4 and an increase in Q2, revealing that the nanoparticles induced late apoptosis in *C. auris*. Furthermore, results also depict that cells exposed to sub-inhibitory concentrations of the Ag–Fe NPs showed early apoptosis. In contrast, as late apoptosis and necrosis were observed when cells were exposed to higher concentrations of the Ag–Fe NPs.

During apoptosis, PS is exposed to the outer side of the plasma membrane, which is not the scenario in healthy growing cells. An early apoptotic condition is very well judged by the process of PS externalization. Previous studies have established the efficacy of Ag NPs against *C. albicans* [75,76,77,78] by destabilizing mitochondrial membrane potential and pore formation, which results in the leakage of ions and restorative materials [79]. This leakage causes ultrastructural changes and thereby induces cellular apoptosis [70,80]. Additionally, naphthofuranquinones, carvacrol and synthetic MCh-AMP1 are a few such compounds that exert anti-*Candida* activity by damaging the plasma membrane, PS externalization, mitochondrial membrane depolarization, ROS production, DNA fragmentation and finally, apoptosis [81,82,83]. Therefore, the present finding is in agreement with the former results suggesting Ag–Fe NPs are directly linked to apoptosis, and with this mode of action, it inhibits the growth and survival of *C. auris* cells.

#### 3.2.5. Cell Cycle Arrest

**Figure 9:**
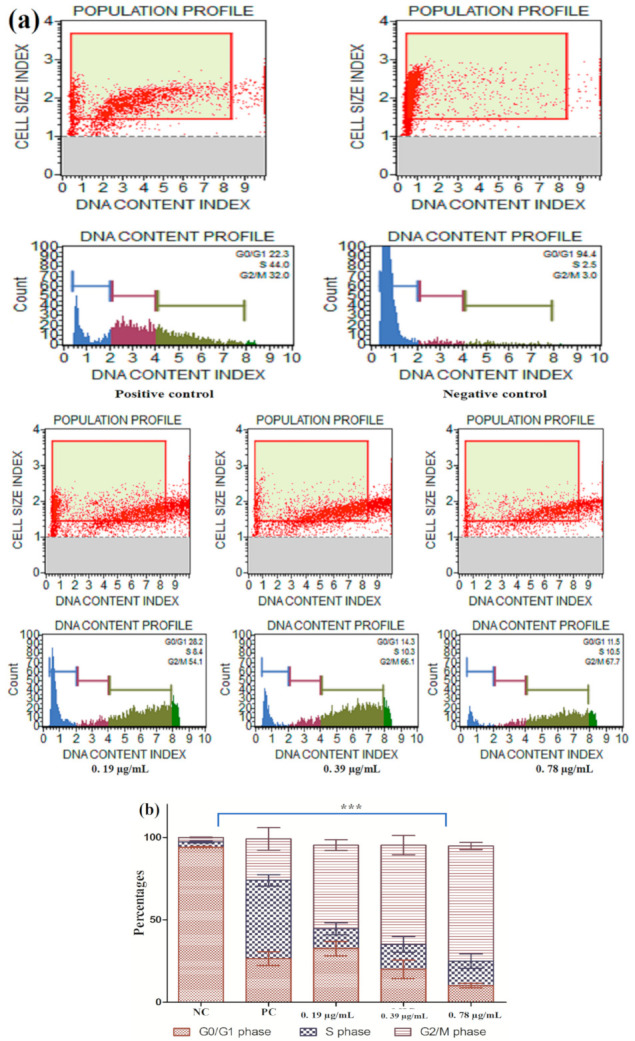
Analysis of cell cycle arrest in *C. auris*. (**a**) Representative images of cell cycle progression and cell size index in *C. auris* MRL6057 after exposure to ½ MIC, MIC and MFC values of the Ag–Fe NPs. Cells exposed to H_2_O_2_ were the positive control (PC), whereas untreated cells were the negative control (NC). (**b**) Average values were obtained for exposed and unexposed *C. auris* cell cycles, representing the effects of Ag–Fe NPs on different stages of cell cycle. *** *p* ≤ 0.05.

The results in the above sections reported the Ag–Fe NPs induced apoptosis, which can be related to DNA damage. It is well known that DNA damaged cells cannot enter the cell cycle to prevent mutations. Therefore, we determined the effect of Ag–Fe NPs on cell cycle arrest in *C. auris*. In healthy growing cells, around 94.1% of the cells were in the G0/G1 phase; S phase and G2/M phase had 3.1% and 2.75% of cells respectively. In 10 mM H_2_O_2_-exposed positive control cells, G0/G1 phase had 26.45%; in S and G2/M phases 47.5% and 25.2% of cells were recorded respectively. Exposure of *C. auris* MRL6057 cells to the Ag–Fe NPs resulted in cell cycle arrest at G2/M phase (Figure 9). At a concentration of 0.195 µg/mL the distribution of *C. auris* in the cell cycle was 32.55%, 12% and 50.8% in G0/G1, S and G2/M phases, respectively. Additionally, at 0.39 µg/mL of Ag–Fe NPs, the percentage of cells in the G2/M phase increased to 60.25%, whereas the percentages of cells in the S and G0/G1 phases were 15.1% and 20%, respectively. However, at a still higher concentration (0.78 µg/mL) the percentage of cells in G2/M phase was raised to 69.9%, whereas there was a slow increase in the percentage of cells in S phase (14.9%), and the percentage of cells in G0/G1 phase decreased rapidly to 10.05%. Overall, the results revealed that the Ag–Fe NPs arrested the cell cycle in G2/M phase. Consequently, the Ag–Fe NPs had a significant effect on the progression of the cell cycle in *C. auris*. The cells were mainly arrested in G2/M phase in a dose-dependent manner with increased arrest in G2/M phase at higher concentrations of the Ag–Fe NPs.

The modulatory effect of Ag–Fe NPs on the cell cycle in *C. auris* MRL6057 further strengthens anti-candidal potency of these NPs. Since the cell cycle is a vital process required for cell proliferation, if the percentages of cells present in different phases of the cell cycle are distorted compared to the healthy growing cells, this will result in cell cycle arrest. In this regard, DNA content change was estimated during different cell cycle phases by quantitative estimation of a single cell, and cells can be differentiated in different phases of the cell cycle based on the fluorescence intensity produced by DNA labeled with PI, which is directly proportional to a particular phase in the cell cycle.

Our results agree with several previous studies reporting the cell cycle arrest in the G2/M phase when *Candida* cells were treated with different external agents. Ag NPs have been reported to arrest the cell cycle during the G2/M phase in *C. albicans* [81], resulting in G2/M-phase-mediated apoptosis [84]. Additionally, crambescidin-816, crambescidin-089 and clioquinol, have been previously reported to arrest the cell cycle at the G2/M phase in *Candida* species and *Saccharomyces cerevisiae* [85]. Impairment of the cell cycle triggers fungal cell morphology changes that increase *Candida* cells’ recognition by the host immune system [86]. Therefore, Ag–Fe NPs directly target the *C. auris* cell cycle and enhance its recognition by the immune cells, further strengthening their candidature for anti-*Candida* treatment.

#### 3.2.6. Enzyme Assays

**Figure 10:**
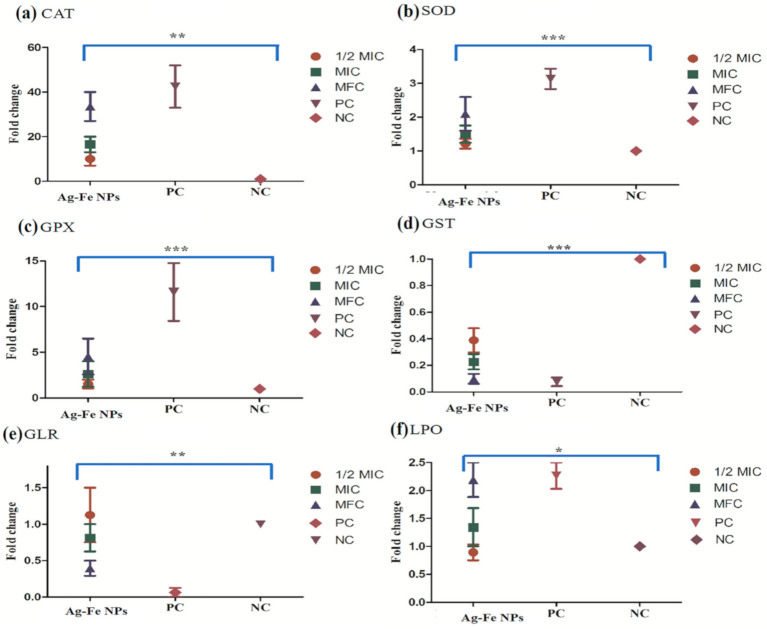
Antioxidant enzyme activity of *C. auris*. The effects of 0.19 µg/mL (½ MIC), 0.39 µg/mL (MIC) and 0.78 µg/mL (MFC) on the activity of different antioxidant enzymes in *C. auris* MRL6057. (**a**–**f**) represent catalase, superoxide dismutase, glutathione peroxidise, glutathione transferase, glutathione reductase and lipid peroxidation respectively. * *p* ≤ 0.001; ** *p* ≤ 0.005; *** *p* ≤ 0.05.

Assessment of changes in the activity of different vital antioxidant enzymes following exposure to Ag–Fe NPs was performed. The changes were also correlated with oxidative stress response in *C. auris* strain. The results obtained for all the enzyme activity and LPO are represented in Figure 10.

Catalase is considered a primary oxidative defense enzyme, and with an increasing concentration of Ag–Fe NPs, the activity of the enzyme increased. The average enzymatic activity for healthy growing cells was 0.091 μmol of H_2_O_2_ consumed/min. Compared with the untreated control, the fold increases in enzyme activity were calculated as 10, 16.5 and 33.5 at ½ MIC, MIC and MFC, whereas the positive control showed a 42.5 folds increase. Similarly, SOD activity also showed an increasing trend after exposure to Ag–Fe NPs. The untreated cells were recorded with an average enzymatic activity of 100.49 units/mL, which gradually increased when cells were exposed to higher concentrations of Ag–Fe NPs. The relative fold increases observed at ½ MIC, MIC and MFC were 1.21, 1.5 and 2.09, respectively; as for the positive control, the fold-increase was 3.13. In the case of GPx, an increasing trend was observed. The untreated control had an average enzyme activity of 3.86 × 10^−7^ µmol of NADPH oxidized/min. However, after exposure, the activity was found to increase 1.53, 2.62 and 4.54 folds with the respective concentrations of Ag–Fe NPs; and for the positive control, the fold increase was 11.59. In contrast to primary enzymes, there was a decline in the enzyme activity of secondary enzymes (GLR and GST) when cells were exposed to higher concentrations of Ag–Fe NPs. For healthy cells, GLR and GST values were 2 × 10^−5^ μmol of NADPH oxidized/min and 2 × 10^−4^ μmol of NADPH oxidized/min, respectively. However, the values went down to 1.12, 0.81, 0.39 and 0.06 folds respectively for ½ MIC, MIC, MFC and positive control for GLR; and 0.09, 0.22, 0.38 and 0.07 folds respectively for ½ MIC, MIC, MFC and positive control for GST. The rate of formation of TBARS determined lipid peroxidation, and exposure to Ag–Fe NPs showed a gradual increase in the rate TBARS formation compared to the untreated control. The fold increases calculated for TBARS formed at ½ MIC, MIC and MFC were 0.89, 1.34 and 2.19, respectively. Ag–Fe NPs were found to modulate oxidative stress parameters in *C. auris*, which was demonstrated from the enzymatic activity of different defense enzymes and was further supported by the increased LPO levels. In *C. albicans* the antioxidant system controls antifungal resistance, morphogenesis and immune coping mechanisms [87]. Oxidative stress is an outcome of elevated levels of reactive oxidant species (ROS) due to foreign elements, such as drugs that can lead to membrane depolarization. Oxidative stress triggers lipid oxidation and DNA disorganization, leading to structural and molecular modifications; these are unique cell death predictors.

The cell’s ROS generation is always balanced with various antioxidant defenses, including enzymatic and non-enzymatic scavengers—mainly CAT, SOD, GPX, GST and GSH. ROS is considered important for controlling normal biochemical functions involved in survival, such as proliferation, cell cycle, differentiation, and cell death [88]. However, if the antioxidant detoxification mechanism fails to keep up ROS within a tolerable level, then the increased cellular level of ROS can trigger oxidative stress within the cell. High cellular concentrations of ROS can destroy proteins, nucleic acids, and membranes, which can further induce cellular apoptosis. ROS also has a crucial role in cell signaling and regulating cellular apoptosis mediated by mitochondria, death receptors and the endoplasmic reticulum [89]. Therefore, the present study strongly supports the fact that Ag–Fe NPs results in oxidative stress in *C. auris*, which is then complimented by cellular apoptosis and cell cycle arrest within the cells.

#### 3.2.7. Hemolytic Activity of Ag–Fe NPs

Toxicity assessment is an essential step towards antifungal drug development. Therefore, it is imperative to look for compounds that are highly specific for *Candida*. In the progression of our study, Ag–Fe NPs showed intense anti-*Candida* activity by generating oxidative stress, inducing cellular apoptosis, and causing cell cycle arrest in *C. auris* isolates. The hemolytic activity of the Ag–Fe NPs using horse erythrocytes was tested. As expected, no lysis was observed in untreated cells, whereas 100% hemolysis was caused by triton X. In contrast to controls, Ag–Fe NPs at inhibitory and sub-inhibitory concentrations caused far less hemolysis (2.93–7%), whereas at higher concentrations (MFC), a rate of 12.93% hemolysis was observed. These results confirmed that Ag–Fe NPs are not toxic even at a double the MIC, supporting its use for animal studies.

It is also important to note that the structure, size, and shape of nanoparticles determine their cytotoxicity; therefore, it is essential to enhance their stability and biocompatibility during their preparation [90]. For instance, researchers found that purification of golden nanoparticles coated with glutathione (Au-GSH NPs) by ultracentrifugation during the different steps and surface modifications resulted in highly reduced cellular toxicity to human cell lines [91]. In this study, several steps have been taken during the pre-and post-synthesis processes of these nanoparticles to reduce their cellular toxicity, as is evident from the hemolytic assay results. However, studies with human cell lines and animal models will further verify the safe use of Ag–Fe NPs.

## 4. Conclusions

The present study demonstrated that Ag–Fe NPs affect *C. auris* growth and survival negatively. It was confirmed that the cumulative effects of Ag–Fe NPs are related to the modulation of crucial antioxidant enzymes, resulting in the generation of oxidative stress and the arrest of the cell cycle in the G2/M phase, leading to programmed cell death in *C. auris* cells. Moreover, Ag–Fe NPs promotes cellular apoptosis in *C. auris* through the generation of oxidative stress within the cells, arresting the cell cycle in G2/M phase, disrupting the mitochondrial integrity and triggering the release of cytochrome c from the cell. Therefore, we conclude that Ag–Fe NPs have intense anti-*Candida* activity against MDR *C. auris* strains, and the nontoxic nature of Ag–Fe NPs makes them safe for stage II in vivo studies. Hence, they can be used as a potential alternative for antifungal chemotherapy against pathogenic yeasts.

## Figures and Tables

**Table 1:** Antifungal activity of Ag–Fe NPs against different *Candida auris* isolates (*n* = 25).

*C. auris* Isolates	Ag–Fe NPs (µg/mL)		Amphoterecin B (µg/mL)	
	MIC	MFC	MIC	MFC
MRL 6326	0.19	0.39	0.25	0.5
MRL 6183	0.19	0.39	0.25	0.5
MRL 4888	0.39	0.78	1.0	2.0
MRL 6015	0.19	0.39	0.25	0.5
MRL 6333	0.19	0.39	0.5	1.0
MRL 4587	0.19	0.39	0.5	0.5
MRL 6334	0.19	0.39	0.5	2.0
MRL 3785	0.19	0.39	0.125	0.5
MRL 6059	0.19	0.39	0.5	1.0
MRL 4000	0.39	0.78	2.0	4.0
MRL 6065	0.39	0.78	1.0	2.0
MRL 2921	0.39	0.78	2.0	4.0
MRL 6125	0.19	0.39	0.25	0.5
MRL 6338	0.19	0.39	0.25	0.5
MRL 3499	0.19	0.39	0.5	1.0
MRL 6194	0.19	0.39	0.25	0.5
MRL 6005	0.39	0.78	1.0	2.0
MRL 6057	0.39	0.78	4.0	8.0
MRL 5762	0.39	0.78	2.0	4.0
MRL 6173	0.19	0.39	0.25	0.5
MRL 5765	0.39	0.78	2.0	4.0
MRL 2397	0.39	0.78	1.0	2.0
MRL 5418	0.19	0.39	0.5	1.0
MRL 6277	0.19	0.39	0.5	1.0
MRL 6339	0.19	0.39	0.5	1.0

## Data Availability

All relevant data are within the manuscript.

## Supplementary Materials

The following are available online at https://www.mdpi.com/2076-3921/10/2/182/s1, Figure S1: Disc diffusion assay of Ag–Fe NPs against *C. auris* MRL6057. MIC and MFC represent 0.39 and 0.78 µg/mL of Ag–Fe NPs, whereas control discs were impregnated with 1% DMSO. AmB represents discs impregnated with 2 µg/mL of amphotericin B.

## References

[1] Kordalewska M., Perlin D.S. (2019). Identification of drug resistant *Candida auris*. Front. Microbiol..

[2] Zamith-Miranda D., Heyman H.M., Cleare L.G., Couvillion S.P., Clair G.C., Bredeweg E.L., Gacser A., Nimrichter L., Nakayasu E.S., Nosanchuk J.D. (2019). Multi-omics signature of *Candida auris*, an emerging and multidrug-resistant pathogen. mSystems.

[3] Crane R.A., Scott T.B. (2012). Nanoscale zero-valent iron: Future prospects for an emerging water treatment technology. J. Hazard. Mater..

[4] Sivamaruthi B.S., Ramkumar V.S., Archunan G., Chaiyasut C., Suganthy N. (2019). Biogenic synthesis of silver palladium bimetallic nanoparticles from fruit extract of Terminalia chebula—In vitro evaluation of anticancer and antimicrobial activity. J. Drug Deliv. Sci. Technol..

[5] Kamli M.R., Srivastava V., Hajrah N.H., Sabir J.S.M., Hakeem K.R., Ahmad A., Malik M.A. (2021). Facile Bio-Fabrication of Ag-Cu-Co Trimetallic Nanoparticles and Its Fungicidal Activity against Candida auris. J. Fungi.

[6] George J.M., Priyanka R.N., Mathew B. (2020). Bimetallic Ag–Au nanoparticles as pH dependent dual sensing probe for Mn (II) ion and ciprofloxacin. Microchem. J..

[7] Song Y., Lin Y., Chu X., Tang J., Xu S. (2020). Facile synthesis of supported AuNi and PtNi bimetallic nanomaterials and their enhanced catalytic properties. J. Mater. Res. Technol..

[8] Ferrando R., Jellinek J., Johnston R.L. (2008). Nanoalloys: From theoryto applications of alloy clusters and nanoparticles. Chem Rev..

[9] Wang D.S., Li Y.D. (2011). Bimetallic nanocrystals: Liquid-phasesynthesis and catalytic applications. Adv. Mater..

[10] Kim D., Resasco J., Yu Y., Asiri A.M., Yang P.D. (2014). Synergisticgeometric and electronic effects for electrochemicalreduction of carbon dioxide using gold-copper bimetallicnanoparticles. Nat. Commun..

[11] Ataee-Esfahani H., Wang L., Nemoto Y., Yamauchi Y. (2010). Synthesisof bimetallic Au@Pt nanoparticles with Au core andnanostructured Pt shell toward highly activeelectrocatalysts. Chem. Mater..

[12] Cui Y., Ren B., Yao J.L., Gu R.A., Tian Z.Q. (2006). Synthesis of Ag core Aushell bimetallic nanoparticles for immunoassay based onsurface-enhanced Raman spectroscopy. J. Phys. Chem. B.

[13] Lim B., Jiang M.J., Camargo P.H.C., Cho E.C., Tao J., Lu X.M., Zhu Y., Xia Y. (2009). Pd-Pt bimetallic nanodendrites with high activity for oxygenreduction. Science.

[14] Yi W.Z., Yuan W.T., Meng Y., Zou S.H., Zhou Y.H., Hong W., Che J., Hao M., Ye B., Xiao L. (2017). Arational solid-state synthesis of supported Au-Ni bimetallicnanoparticles with enhanced activity for gas-phase selectiveoxidation of alcohols. ACS Appl. Mater. Interfaces.

[15] Rodriguez-Gonzalez B., Burrows A., Watanabe M., Kiely C.J., Liz-Marzan L.M. (2005). Multishell bimetallic AuAg nanoparticles:synthesis, structure and optical properties. J. Mater. Chem..

[16] Yallappa S., Manjanna J., Dhananjaya B.L. (2015). Phytosynthesis of stable Au, Ag and Au-Ag alloy nanoparticles using J. sambac leaves extract, and their enhanced antimicrobial activity in presence of organic antimicrobials. Spectrochim. Acta A Mol. Biomol. Spectrosc..

[17] Zhang G., Du M., Li Q., Li X., Huang J., Jiang X., Sun D. (2013). Green synthesis of Au–Ag alloy nanoparticles using Cacumen platycladi extract. RSC Adv..

[18] Rane A.V., Kanny K., Abitha V.K., Thomas S. (2018). Methods for synthesis of nanoparticles and fabrication of nanocomposites. Synth. Inorg. Nanomater..

[19] Dhand C., Dwivedi N., Loh X.J., Ying A.N.J., Verma N.K., Beuerman R.W., Lakshminarayanan R., Ramakrishna S. (2015). Methods and strategies for the synthesis of diverse nanoparticles and their applications: A comprehensive overview. Rsc Adv..

[20] Khan Z., Al-Thabaiti S.A., Obaid A.Y., Malik M.A., Khan M.N., Khan T.A. (2016). Cobalt@ silver bimetallic nanoparticles: Solution based seedless surfactant assisted synthesis, optical properties, and morphology. J. Mol. Liq..

[21] Alzahrani S.A., Al-Thabaiti S.A., Shamsan Al-Arjan W., Malik M.A., Khan Z. (2017). Preparation of ultra-long α-MnO_2_ and Ag@MnO_2_ nanoparticles by seedless approach and their photocatalytic performance. J. Mol. Str..

[22] Gahlawat G., Choudhury A.R. (2019). A review on the biosynthesis of metal and metal salt nanoparticles by microbes. RSC Adv..

[23] Akhtar M.S., Panwar J., Yun Y.S. (2013). Biogenic synthesis of metallic nanoparticles by plant extracts. ACS Sustain. Chem. Eng..

[24] Roy A., Bulut O., Some S., Mandal A.K., Deniz Yilmaz M. (2019). Green synthesis of silver nanoparticles: Biomolecule-nanoparticle organizations targeting antimicrobial activity. RSC Adv..

[25] Alshehri A.A., Malik M.A. (2020). Facile One-pot biogenic synthesis of Cu-Co-Ni trimetallic nanoparticles for enhanced photocatalytic dye degradation. Catalysts.

[26] Singh P., Kim Y.J., Zhang D., Yang D.C. (2016). Biological synthesis of nanoparticles from plants and microorganisms. Trends Biotechnol..

[27] Ahmad A., Senapati S., Khan M.I., Kumar R., Sastry M. (2003). Extracellular biosynthesis of monodisperse gold nanoparticles by a novel extremophilic actinomycete, *Thermomonospora* sp. Langmuir.

[28] Ahmad A., Mukherjee P., Senapati S., Mandal D., Khan M.I., Kumar R., Sastry M. (2003). Extracellular biosynthesis of silver nanoparticles using the fungus *Fusarium oxysporum*. Colloids Surf. B Biointerfaces.

[29] Ahmed S., Ahmad M., Swami B.L., Ikram S. (2016). Green synthesis of silver nanoparticles using Azadirachta indica aqueous leaf extract. J. Radiat. Res. Appl. Sci..

[30] Folorunso A., Akintelu S., Oyebamiji A.K., Ajayi S., Abiola B., Abdusalam I., Morakinyo A. (2019). Biosynthesis, characterization and antimicrobial activity of gold nanoparticles from leaf extracts of Annona muricata. J. Nanostr. Chem..

[31] AbdelHamid A.A., Al-Ghobashy M.A., Fawzy M., Mohamed M.B., Abdel-Mottaleb M.M. (2013). Phytosynthesis of Au, Ag, and Au–Ag bimetallic nanoparticles using aqueous extract of sago pondweed (*Potamogeton pectinatus* L.). ACS Sustain. Chem. Eng..

[32] Gopalakrishnan R., Loganathan B., Raghu K. (2015). Green synthesis of Au–Ag bimetallic nanocomposites using Silybum marianum seed extract and their application as a catalyst. RSC Adv..

[33] Vilas V., Philip D., Mathew J. (2016). Biosynthesis of Au and Au/Ag alloy nanoparticles using Coleus aromaticus essential oil and evaluation of their catalytic, antibacterial and antiradical activities. J. Mol. Liq..

[34] Sharma C., Ansari S., Ansari M.S., Satsangee S.P., Srivastava M.M. (2020). Single-step green route synthesis of Au/Ag bimetallic nanoparticles using clove buds extract: Enhancement in antioxidant bio-efficacy and catalytic activity. Mater. Sci. Eng. C.

[35] Weng X., Guo M., Luo F., Chen Z. (2017). One-step green synthesis of bimetallic Fe/Ni nanoparticles by eucalyptus leaf extract: Biomolecules identification, characterization and catalytic activity. Chem. Eng. J..

[36] Song J.Y., Kim B.S. (2009). Rapid biological synthesis of silver nanoparticles using plant leaf extracts. Bioprocess Biosyst. Eng..

[37] Al-Asfar A., Zaheer Z., Aazam E.S. (2018). Eco-friendly green synthesis of Ag-Fe bimetallic nanoparticles: Antioxidant, antimicrobial and photocatalytic degradation of bromothymol blue. J. Photochem. Photobiol. B.

[38] Venugopal K., Ahmad H., Manikandan E., Thanigai A.K., Kavitha K., Moodley M.K., Rajagopal K., Balabhaskar R., Bhaskar M. (2017). The impact of anticancer activity upon *Beta vulgaris* extract mediated biosynthesized silver nanoparticles (Ag-NPs) against human breast (MCF-7), lung (A549) and pharynx (Hep-2) cancer cell lines. J. Photochem. Photobiol. B.

[39] Parameshwaran R., Kalaiselvam S., Jayavel R. (2013). Green synthesis of silver nanoparticles using *Beta vulgaris*: Role of process conditions on size distribution and surface structure. Mater. Chem. Phys..

[40] Foroozandeh P., Aziz A.A. (2018). Insight into cellular uptake and intracellular trafficking of nanoparticles. Nanoscale Res. Lett..

[41] Zhu M., Nie G., Meng H., Xia T., Nel A., Zhao Y. (2012). Physicochemical properties determine nanomaterial cellular uptake, transport, and fate. Acc. Chem. Res..

[42] Nel A.E., Mädler L., Velegol D., Xia T., Hoek E.M., Somasundaran P. (2009). Understanding biophysicochemical interactions at the nano–bio interface. Nat. Mater..

[43] Panariti A., Miserocchi G., Rivolta I. (2012). The effect of nanoparticle uptake on cellular behavior: Disrupting or enabling functions?. Nanotechnol. Sci. Appl..

[44] Rejman J., Oberle V., Zuhorn I., Hoekstra D. (2004). Size-dependent internalization of particles via the pathways of clathrin-and caveolae-mediated endocytosis. Biochem. J..

[45] Lee S.Y., Chen H.F., Yeh Y.C., Xue Y.P., Lan C.Y. (2019). The transcription factor Sfp1 regulates the oxidative stress response in *Candida albicans*. Microorganisms.

[46] Redza-Dutordoir M., Averill-Bates D.A. (2016). Activation of apoptosis signaling pathways by reactive oxygen species. Biochim. Biophys. Acta.

[47] Clinical and Laboratory Standards Institute (2008). Reference Method for Broth Dilution Antifungal Susceptibility Testing of Yeast, Approved Standard M27-A3.

[48] Gutiérrez J.A., Caballero S., Diaz L.A., Guerrero M.A., Ruiz J., Ortiz C.C. (2018). High antifungal activity against candida species of monometallic and bimetallic nanoparticles synthesized in nanoreactors. ACS Biomater. Sci. Eng..

[49] Markova Z., Siskova K.M., Filip J., Cuda J., Kolar M., Safarova K., Medrik I., Zboril R. (2013). Air stable magnetic bimetallic fe–ag nanoparticles for advanced antimicrobial treatment and phosphorus removal. Environ. Sci. Technol..

[50] Lozhkomoev A.S., Lerner M.I., Pervikov A.V., Kazantsev S.O., Fomenko A.N. (2018). Development of Fe/Cu and Fe/Ag Bimetallic nanoparticles for promising biodegradable materials with antimicrobial effect. Nanotechnol. Russ..

[51] Lone S.A., Wani M.Y., Fru P., Ahmad A. (2020). Cellular apoptosis and necrosis as therapeutic targets for novel eugenol tosylate congeners against *Candida albicans*. Sci. Rep..

[52] Ahmad A., Khan A., Manzoor N. (2013). Reversal of efflux mediated antifungal resistance underlies synergistic activity of two monoterpenes with fluconazole. Eur. J. Pharm..

[53] Yun D.G., Lee D.G. (2016). Silibinin triggers yeast apoptosis related to mitochondrial Ca^2+^ influx in *Candida albicans*. Int. J. Biochem. Cell. Biol..

[54] Khan A., Ahmad A., Ahmad Khan L., Padoa C.J., Van Vuuren S., Manzoor N. (2015). Effect of two monoterpene phenols on antioxidant defense system in *Candida albicans*. Microb. Pathog..

[55] Yousuf S., Ahmad A., Khan A., Manzoor N., Khan L.A. (2010). Effect of diallyldisulphide on an antioxidant enzyme system in *Candida* species. Can. J. Microbiol..

[56] Maras B., Angiolella L., Mignogna G., Vavala E., Macone A., Colone M., Pitari G., Stringaro A., Dupré S., Palamara A.T. (2014). Glutathione metabolism in *Candida albicans* resistant strains to fluconazole and micafungin. PLoS ONE.

[57] Zhang D., Lanier S.M., Downing J.A., Avent J.L., Lum J., McHale J.L. (2008). Betalain pigments for dye-sensitized solar cells. J. Photochem. Photobiol. A Chem..

[58] Lankoff A., Sandberg W.J., Wegierek-Ciuk A., Lisowska H., Refsnes M., Sartowska B., Schwarze P.E., Meczynska-Wielgosz S., Wojewodzka M., Kruszewski M. (2012). The effect of agglomeration state of silver and titanium dioxide nanoparticles on cellular response of HepG2, A549 and THP-1 cells. Toxicol. Lett..

[59] Argentiere S., Cella C., Cesaria M., Milani P., Lenardi C. (2016). Silver nanoparticles in complex biological media: Assessment of colloidal stability and protein corona formation. J. Nanopart. Res..

[60] Bae E., Lee B.-C., Kim Y., Choi K., Yi J. (2013). Effect of agglomeration of silver nanoparticle on nanotoxicity depression. Korean J. Chem. Eng..

[61] Peña-González C.E., Pedziwiatr-Werbicka E., Martín-Pérez T., Szewczyk E.M., Copa-Patiño J.L., Soliveri J., Pérez-Serrano J., Gómez R., Bryszewska M., Sánchez-Nieves J. (2017). Antibacterial and antifungal properties of dendronized silver and gold nanoparticles with cationic carbosilane dendrons. Int. J. Pharm..

[62] Selvaraj M., Pandurangan P., Ramasami N., Rajendran S.B., Sangilimuthu S.N., Perumal P. (2014). Highly potential antifungal activity of quantum-sized silver nanoparticles against *Candida albicans*. Appl. Biochem. Biotechnol..

[63] Parveen S., Wani A.H., Shah M.A., Devi H.S., Bhat M.Y., Koka J.A. (2018). Preparation, characterization and antifungal activity of iron oxide nanoparticles. Microb. Pathog..

[64] Loza K., Heggen M., Epple M. (2020). Synthesis, structure, properties, and applications of bimetallic nanoparticles of noble metals. Adv. Funct. Mater..

[65] Giner-Casares J.J., Henriksen-Lacey M., Coronado-Puchau M., Liz-Marzán L.M. (2016). Inorganic nanoparticles for biomedicine: Where materials scientists meet medical research. Mater. Today.

[66] Li Y., Yang D., Wang S., Li C., Xue B., Yang L., Shen Z., Jin M., Wang J., Qiu Z. (2018). The detailed bactericidal process of ferric oxide nanoparticles on *E. coli*. Molecules.

[67] Arakha M., Pal S., Samantarrai D., Panigrahi T.K., Mallick B.C., Pramanik K., Mallick B., Jha S. (2015). Antimicrobial activity of iron oxide nanoparticle upon modulation of nanoparticle-bacteria interface. Sci. Rep..

[68] Dlugaszewska J., Dobrucka R. (2019). Effectiveness of biosynthesized trimetallic Au/Pt/Ag nanoparticles on planktonic and biofilm *Enterococcus faecalis* and *Enterococcus faecium* forms. J. Clust. Sci..

[69] Akter M., Sikder M.T., Rahman M.M., Ullah A.K.M.A., Hossain K.F.B., Banik S., Hosokawa T., Saito T., Kurasaki M. (2017). A systematic review on silver nanoparticles-induced cytotoxicity: Physicochemical properties and perspectives. J. Adv. Res..

[70] Hwang I.S., Lee J., Hwang J.H., Kim K.J., Lee D.G. (2012). Silver nanoparticles induce apoptotic cell death in *Candida albicans* through the increase of hydroxyl radicals. FEBS J..

[71] Zhu M.T., Wang Y., Feng W.Y., Wang B., Wang M., Ouyang H., Chai Z.F. (2010). Oxidative stress and apoptosis induced by iron oxide nanoparticles in cultured human umbilical endothelial cells. J. Nanosci. Nanotechnol..

[72] Huttemann M., Pecina P., Rainbolt M., Sanderson T.H., Kagan V.E., Samavati L., Doan J.W., Lee I. (2011). The multiple functions of cytochrome c and their regulation in life and death decisions of the mammalian cell: From respiration to apoptosis. Mitochondrion.

[73] Adrain C., Martin S.J. (2001). The mitochondrial apoptosome: A killer unleashed by the cytochrome seas. Trends Biochem. Sci..

[74] Jia C., Zhang J., Yu L., Wang C., Yang Y., Rong X., Xu K., Chu M. (2019). Antifungal activity of coumarin against *Candida albicans* is related to apoptosis. Front. Cell Infect. Microbiol..

[75] Panácek A., Kolár M., Vecerová R., Prucek R., Soukupová J., Krystof V., Hamal P., Zboril R., Kvítek L. (2009). Antifungal activity of silver nanoparticles against *Candida* spp. Biomaterials.

[76] Wady A.F., Machado A.L., Zucolotto V., Zamperini C.A., Berni E., Vergani C.E. (2012). Evaluation of *Candida albicans* adhesion and biofilm formation on a denture base acrylic resin containing silver nanoparticles. J. Appl. Microbiol..

[77] Lara H.H., Romero-Urbina D.G., Pierce C., Lopez-Ribot J.L., Arellano-Jimenez M.J., Jose-Yacaman M. (2015). Effect of silver nanoparticles on *Candida albicans* biofilms: An ultrastructural study. J. Nanobiotechnol..

[78] Monteiro D.R., Takamiya A.S., Feresin L.P., Gorup L.F., De Camargo E.R., Delbem A.C., Henriques M., Barbosa D.B. (2015). Susceptibility of *Candida albicans* and *Candida glabrata* biofilms to silver nanoparticles in intermediate and mature development phases. J. Prosthodont. Res..

[79] Kim K.-J., Sung W.S., Suh B.K., Moon S.-K., Choi J.-S., Kim J.G., Lee D.G. (2009). Antifungal activity and mode of action of silver nanoparticles on *Candida albicans*. Biometals.

[80] Vazquez-Munoz R., Avalos-Borja M., Castro-Longoria E. (2014). Ultrastructural analysis of *Candida albicans* when exposed to silver nanoparticles. PLoS ONE.

[81] Neto J.B.A., Da Silva C.R., Neta M.A.S., Campos R.S., Siebra J.T., Silva R.A.C., Gaspar D.M., Magalhaes H.I., De Moraes M.O., Lobo M.D. (2014). Antifungal activity of Naphthoquinoidal compounds in vitro against fluconazole-resistant strains of different *Candida* species: A special emphasis on mechanisms of action on *Candida tropicalis*. PLoS ONE.

[82] Niu C., Wang C., Yang Y., Chen R., Zhang J., Chen H., Zhuge Y., Li J., Cheng J., Xu K. (2020). Carvacrol induces *Candida albicans* apoptosis associated with Ca^2+^/calcineurin pathway. Front. Cell Infect. Microbiol..

[83] Seyedjavadi S.S., Khani S., Eslamifar A., Ajdary S., Goudarzi M., Halabian R., Akbari R., Zare-Zardini H., Imani Fooladi A.A., Amani J. (2020). The antifungal peptide MCh-AMP1 derived from *Matricaria chamomilla* inhibits *Candida albicans* growth *via* inducing ROS generation and altering fungal cell membrane permeability. Front. Microbiol..

[84] Phillips A.J., Sudbery I., Ramsdale M. (2003). Apoptosis induced by environmental stresses and amphotericin B in *Candida albicans*. Proc. Natl. Acad. Sci. USA.

[85] Rubiolo J.A., Ternon E., López-Alonso H., Thomas O.P., Vega F.V., Vieytes M.R., Botana L.M. (2013). Crambescidin-816 acts as a fungicidal with more potency than crambescidin-800 and -830, inducing cell cycle arrest, increased cell size and apoptosis in *Saccharomyces cerevisiae*. Mar. Drugs.

[86] Stefanini I., Rizzetto L., Rivero D., Carbonell S., Gut M., Heath S., Gut I.G., Trabocchi A., Guarna A., Ghazzi N.B. (2018). Deciphering the mechanism of action of 089, a compound impairing the fungal cell cycle. Sci. Rep..

[87] Cannon R.D., Erwin L., Holmes A.R., Niimi K., Koichi T., Niimi M., Monk B.C. (2007). *Candida albicans* drug resistance e another way to cope with stress. Microbiology.

[88] Covarrubias L., Hernandez-Garcia D., Schnabel D., Salas-Vidal E., Castro-Obregon S. (2008). Function of reactive oxygen species during animal development: Passive or active?. Dev. Biol..

[89] Halliwell B. (2011). Free radicals and antioxidants—Quo vadis?. Trends Pharmacol. Sci..

[90] Ficociello G., De Caris M.G., Trillò G., Cavallini D., Sarto M.S., Uccelletti D., Mancini P. (2018). Anti-Candidal activity and in vitro cytotoxicity assessment of graphene nanoplatelets decorated with zinc oxide nanorods. Nanomaterials.

[91] Harper B., Sinche F., Ho Wu R., Gowrishankar M., Marquart G., Mackiewicz M., Harper S.L. (2014). The Impact impact of surface ligands and synthesis method on the toxicity of Glutathione-coated gold nanoparticles. Nanomaterials.

