# Supplementary material for: Phytogenic Fabrication of Ag–Fe Bimetallic Nanoparticles for Cell Cycle Arrest and Apoptosis Signaling Pathways in Candida auris by Generating Oxidative Stress
Source: Antioxidants (Basel). 2021 Jan 27;10(2):182. doi: 10.3390/antiox10020182 (PMC7910930; doi:10.3390/antiox10020182)
Supplement: Supplementary file 1 [file antioxidants-10-00182-s001.pdf]

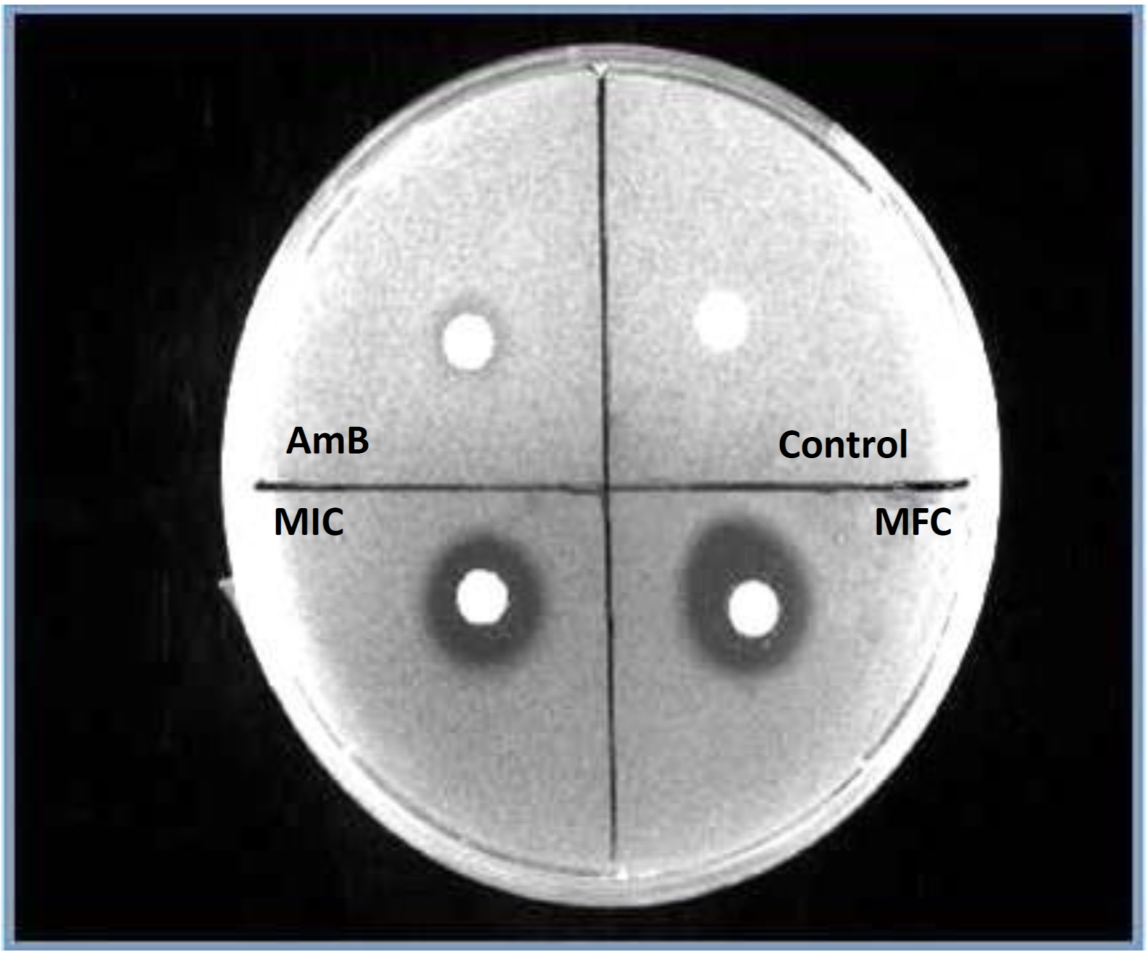


**Figure S1.** Disc diffusion assay of Ag–Fe NPs against *C. auris* MRL6057. MIC and MFC represent 0.39 and 0.78 μg/mL of Ag–Fe NPs, whereas control discs were impregnated with 1% DMSO. AmB represents discs impregnated with 2 μg/mL of amphoterecin B.
